# An FPGA Platform for Real-Time Simulation of Spiking Neuronal Networks

**DOI:** 10.3389/fnins.2017.00090

**Published:** 2017-02-28

**Authors:** Danilo Pani, Paolo Meloni, Giuseppe Tuveri, Francesca Palumbo, Paolo Massobrio, Luigi Raffo

**Affiliations:** ^1^EOLab - Microelectronics and Bioengineering Lab, Department of Electrical and Electronic Engineering, University of CagliariCagliari, Italy; ^2^Information Engineering Unit, PolComIng Department, University of SassariSassari, Italy; ^3^Neuroengineering and Bio-nano Technology Lab, Dibris, University of GenovaGenova, Italy

**Keywords:** FPGA, fixed-point, neural simulator, closed-loop hardware accelerator, real-time

## Abstract

In the last years, the idea to dynamically interface biological neurons with artificial ones has become more and more urgent. The reason is essentially due to the design of innovative neuroprostheses where biological cell assemblies of the brain can be substituted by artificial ones. For closed-loop experiments with biological neuronal networks interfaced with *in silico* modeled networks, several technological challenges need to be faced, from the low-level interfacing between the living tissue and the computational model to the implementation of the latter in a suitable form for real-time processing. Field programmable gate arrays (FPGAs) can improve flexibility when simple neuronal models are required, obtaining good accuracy, real-time performance, and the possibility to create a hybrid system without any custom hardware, just programming the hardware to achieve the required functionality. In this paper, this possibility is explored presenting a modular and efficient FPGA design of an *in silico* spiking neural network exploiting the Izhikevich model. The proposed system, prototypically implemented on a Xilinx Virtex 6 device, is able to simulate a fully connected network counting up to 1,440 neurons, in real-time, at a sampling rate of 10 kHz, which is reasonable for small to medium scale extra-cellular closed-loop experiments.

## 1. Introduction

In the past decades, spiking neuronal networks (SNN) progressively acquired relevance due to possibility to exploit them in several application scenarios. Typical artificial intelligence applications take advantage of the learning capabilities of SNN for classifiers and autonomous controls. Nevertheless, SNN represent a powerful instrument in neuroscience, allowing to simulate living neuronal assemblies trying to gather from the characteristics of a fitted artificial neuronal network clues on the properties of the living tissue (Bonifazi et al., 2013). From this perspective, it is interesting not only the accurate evaluation of the single neuron behavior but, primarily, the study of the emergent properties of the neuronal assembly dynamics (van Pelt et al., 2004). This can be studied through intracellular recordings (single cell models, by voltage clamp techniques) or extracellular recordings (*in vitro* cultures of neurons or cortical implants, by Micro-Electrode Arrays MEAs). In this case, the adoption of SNN software simulators (Brette et al., 2007) is widely accepted and the use of such tools is cumbersome only when it is required to simulate very large networks, because of the explosion of the computational cost and, in turn, of the processing time.

This limitation fostered the research toward the development of hardware accelerators able to carry out such simulations in a shorter time (Cheung et al., 2016), possibly comparable to the time scales of a real evolving neuronal network, in order to enable complex simulations otherwise impossible. With the advancements of the technology, it is possible to look at such hardware simulators as enabling tools for new neuroscience experiments. For instance, closed-loop electrophysiological systems are characterized by the tight interaction between the living neuronal tissue and a controlling electronic unit able to interact with it through sensing and stimulation (Le Masson et al., 2002). Compared to open-loop experiments, closed-loop ones enable the study of both the input and output side of the neuronal assembly at the same time (Rolston et al., 2010). The largest part of systems for closed-loop experiments aim to study this aspect by means of an integration between a sensing interface, simple and abstracted computational models and a stimulation interface. Examples of interfacing with muscles for real-time control have been already presented in the literature (Zbrzeski et al., 2016). One aspect, paving the way to the development of neuroprostheses, would be the direct interface of a population of living neurons to an artificial SNN in order to evaluate the capability of the living system to interact with the simulated one (Bonifazi et al., 2013). This poses severe constraints on the hardware implementation of the SNN, particularly for everything concerning the timing, which is no more a matter of performance improvement over a simple PC implementation, but an aspect connected to the feasibility of the neuroprosthetic approach. Examples of the integration of living neurons into artificial SNN can be already found in the literature (Nawrot et al., 2003).

The goal of this paper is to describe and validate a scalable and modular hardware architecture to simulate the dynamics generated by biologically-plausible synthetic neuronal assemblies in real-time. This architecture was completely manually coded and optimized in Verilog Hardware Description Language (HDL) for a single Field Programmable Gate Array (FPGA) chip, thus it can be easily adopted in any lab for small-to-medium SNN sizes. This, along with a fixed-point implementation of the Izhikevich (IZ) neuronal model (Izhikevich, 2003), confers to the architecture a considerable real-time performance up to 0.1 ms, joined to a programmable delay that can be reduced down to such bound. The architecture is parametric in the number of neurons that can be simulated, with limitations imposed by the hardware only in terms of the possibility of fitting the SNN in a commercial FPGA. The long-term goal of the so developed architecture is to bi-directionally interface the SNN with a biological one. Three different experimental scenarios can be envisioned:

- Use the real-time SNN on FPGA as a stimulator. The signals generated by the SNN can be used to trigger the spontaneous dynamics of the biological networks. Different stimulation protocols can be imagined, for example using the onset of the network bursts, or the frequency of the bursts. The possibility to generate a “natural” stimulation can be used to shift the dynamical states of the biological network;
- Connect in a bi-directional way the real-time SNN on FPGA and a biological neuronal assembly. Although closed-loop stimulation experiments have been already performed (Wagenaar et al., 2005; Wallach et al., 2011), such works have the intrinsically drawback to use “artificial stimulation,” i.e., stereotyped stimuli delivered by a controlled stimulator. By means of the proposed architecture, it becomes feasible to deliver stimuli modulated by the intrinsic dynamics.
- In the long term, the real-time SNN could be used as a tool to replace a damaged biological network. In fact, in the last years, researchers started to develop a new family of prostheses applied to the central nervous system (neuroprostheses). As an example, Berger et al. developed a hippocampal prosthesis improving memory function in behaving rats (Berger et al., 2011, 2012).

## 2. Materials and methods

SNNs are more realistic than the conventional neural networks for neuroscientific simulations, taking into consideration not only the neuronal and the synaptic state, but also the concept of time into their operating model. Artificial neurons' firing activity is determined by the evolution of their membrane potential, which follows the model equations. Not only different models produce different firing behaviors, but within the same model it is often possible to tune the parameters so that the artificial neuron is able to reproduce different firing patterns proper of specific cells.

The architecture is currently based on the implementation of the IZ neuronal model (Izhikevich, 2003), which is characterized by an excellent trade-off between computational complexity and biological accuracy, being able to reproduce several spiking patterns by simply tuning its parameters. The proposed version of the architecture implements the model in fixed-point arithmetic, in order to primarily reduce the memory requirements (known to be a limiting factor for hardware SNN) and the latency of the mathematical computations. The architecture is conceived to be a customizable framework, where the neuronal model can be easily replaced while preserving the global structure, and the same holds for the synapses. The main features pursued in the design phase were the low latency, with a real-time timing constraint of 0.1 ms and a programmable delay between firing activity and its reflection on the network activity. In turn, this means that the output sample rate is 10 ksample/s, which is adequate for closed-loop experimental systems.

### 2.1. Izhikevich spiking model

The simple model of spiking neurons proposed by Izhikevich (2003) is composed of a two-dimensional system of ordinary differential equations.

(1) $$\begin{array}{l} \frac{dv}{dt}=0.04v^{2}+5v+140-u-I \end{array}$$

(2) $$\begin{array}{l} \frac{du}{dt}=a\left(bv-u\right) \end{array}$$

*v* is the *membrane potential* of the neuron and it is modeled according to Equation (1), whereas Equation (2) provides the dynamic of *u* that is the *membrane recovery variable*. The term *I*, in Equation (1), is meant to take into account the contribution of the connected nodes to the considered neuron, by means of the sum of the synaptic currents or injected dc-currents. When a spike is fired, meaning *v* has reached its threshold, the following resetting condition is applied:

(3) $$\begin{array}{l} v\geq v_{th}\Rightarrow \left\{ \begin{array}{l} v=c;\;\\ u=u+d\; \end{array}\right. \end{array}$$

Both the membrane potential and the membrane recovery value are normally measured in mV. The IZ spiking model is capable to reproduce several different firing patterns, 20 in the original article (e.g., chattering, fast spiking, low-threshold spiking, etc.) but others are being studied, representing the known types of neo-cortical and thalamic neurons, by tuning the *a*, *b*, *c*, and *d* dimensionless parameters:

- *a* represents the time scale of the recovery variable *u*. Smaller values result in slower recovery;
- *b* represents the sensitivity of the recovery variable *u* to possible sub-threshold fluctuations of the membrane potential *v*. Larger values indicate *v* and *u* are strongly coupled, resulting in possible sub-threshold oscillations and low-threshold spiking dynamics;
- *c* is the after-spike reset value of *v*;
- *d* determines the after-spike reset value of *u*.

### 2.2. Hardware spiking neural network

**Figure 1:**
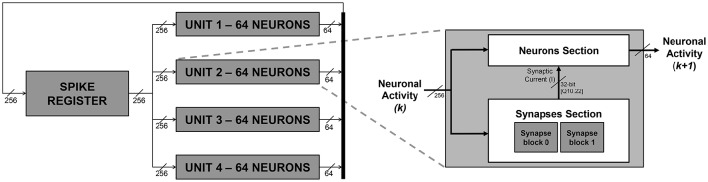
**SNN block diagram (on the left) and ***unit*** block high-level architecture (on the right)**. The numbers reported in the scheme are referred to an exemplary embodiment with a fully connected network of 256 neurons.

In this work, a SNN hardware emulation platform, based on the IZ spiking neuron model, was developed and tested. Figure 1 depicts an overview of the system. The entire neural network is subdivided in *units*, which are the building blocks of the SNN. Each unit produces, according to the IZ spiking neuron model, a sub-set of spikes whose occurrence is stored in the binary *spike register*, which keeps trace of the whole network spiking activity. It is composed of one bit per neuron under emulation. The bit corresponding to a neuron is set high when the neuron has fired a spike. The chosen simulation paradigm is synchronous (or clock-driven), meaning that all neurons are updated at every simulation step, regardless of the spiking activity (Brette et al., 2007). From a macroscopic point of view, each simulation cycle is composed as follows:

1. The *units* process their subset of neurons. They receive the content of the *spike register*, accounting for the whole SNN firing activity (the architectures assumes a fully-connected structure) and, according to the IZ model, they determine which neurons within their subset should fire;
2. The firing activities estimated by the *units* are grouped together by a dedicated *concatenation logic* and sent back to the *spike register*;
3. The *spike register* is updated and represents the updated status of the overall SNN.

Additional details, regarding what happens within the *units* in each simulation step, are provided hereafter in Section 2.2.2. It is worth to notice now that the architecture gives the possibility to set at design time a delay for the spike propagation, exploiting a register chain strategy. The firing activity is propagated back to the units with a delay of several (up to ten) emulation cycles, to mimic a physiological delay of up to 1 ms between firing and its reflection on the network status.

Figure 1 depicts an exemplary instance of the proposed platform, parameterized in order to emulate a network of 256 neurons only for the sake of clarity. The platform instance integrates four *units*, emulating 64 IZ neurons each. According to the signal activity of all the 256 neurons, each unit generates up to 64 spikes per simulation cycle. The four different 64-bit signals, representing each *unit*'s neuronal activity, are concatenated to create a single 256-bit signal and fed back to the *spike register*.

#### 2.2.1. Units: architectural overview

The *units* are responsible for the implementation of Equations (1) and (2) and for estimating the firing activity of the subset of neurons assigned to each of them. Equations (1) and (2) represent two derivatives and they have been implemented in hardware exploiting the finite-difference method, which is a numerical method for solving differential equations by approximating them with finite differences, as specified hereafter:

(4) $$\begin{array}{l} \frac{v\left(k+1\right)-v\left(k\right)}{h}=0.04v{\left(k\right)}^{2}+5v\left(k\right)+140-u\left(k\right)-I \end{array}$$

(5) $$\begin{array}{l} \frac{u\left(k+1\right)-u\left(k\right)}{h}=a\left(bv\left(k\right)-u\left(k\right)\right) \end{array}$$

The smaller is *h* (i.e., the time interval between *k* and *k* + 1) the better Equations (4) and (5), respectively, approximate Equations (1) and (2). In the current implementation, *h* is fixed to 0.1 ms, which turned out to be an excellent compromise between the approximation quality and the overall processing time required to update the status of the entire SNN. Furthermore, such a delta-cycle is compatible with hybrid closed-loop experiments.

Each *unit*, as depicted on the right hand side of Figure 1, is composed of a *Neuron Section* and of a *Synapses Section*, which are, respectively, responsible for computing the overall neuronal activity of the considered *unit* and for determining, for each neuron, the synaptic current (*I*) that has to be subtracted in Equation (4).

**Figure 2:**
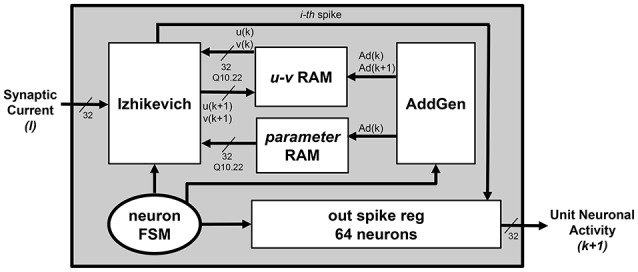
***Unit*****—Overview of the ***Neuron Section*****.

Figure 2 presents an overview of the *Neuron Section* of each single *unit* block. Equations (4) and (5) are physically computed by the *Izhikevich* block, which receives *v*(*k*) and *u*(*k*) values from the *u-v RAM* block, *a*, *b*, *c*, and *d* parameters from the *parameter RAM* block, and finally *I* from the *Synapses Section*. The interactions with the *u-v RAM* and *parameter RAM* blocks, and the synchronization with the *Synapses Section* are controlled by a dedicated Finite State Machine (FSM), the *neuron FSM*. The *neuron FSM* also masters the execution of the different phases of the *Izhikevich* block itself. In terms of library IP module, the latter includes only a multicycle multiplier, which is re-used for all the multiplications in Equations (4) and (5); all the other modules are HDL-coded. In this way, it is possible to save as much hardware resources as possible (by re-using the same processing element rather than instantiating several of them in parallel) and to maintain the operating frequency high (by pipelining the operations and, in turn, breaking down the critical path). The *Izhikevich* block processes one neuron at a time; its output is written on the *out_spike_reg* module, which contains the spikes of all the neurons within the given *unit*. The output of the *Neuron Section* is the updated *unit*'s contribution to the firing activity of the SNN, to be sent to the *concatenation logic*.

**Figure 3:**
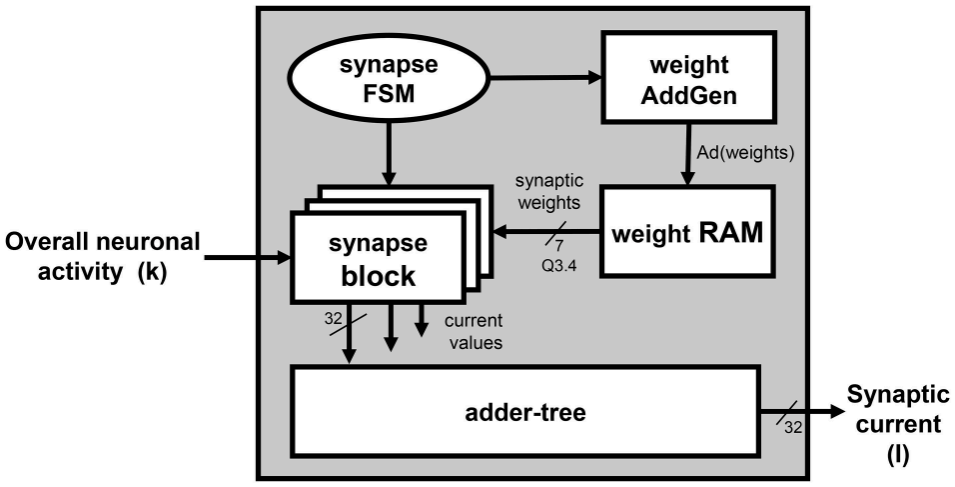
***Unit*****—Overview of the ***Synapses Section*****.

The *Synapses Section* is depicted in the block diagram in Figure 3. The figure shows how the synaptic current is computed on the basis of the overall firing activity (i.e., the content of the *spike register*). It computes the weighted sum of the contribution of the pre-synaptic neurons, connected to the processed one by means of the synapses. Only the synaptic weights associated to those neurons that fired (having a 1 in the corresponding bit of the *spike register*) are eventually accumulated. The *synaptic weights* are provided by the *weight RAM* block. The contribution of each connected neuron is computed by a set of *synapses* blocks, placed in parallel to speed-up the computation since all the incoming neuronal activity has to be scanned to determine the *synaptic weights* associated to the pre-synaptic neurons that fired and, accordingly, retrieve and accumulate them. As an example, the Synapse section of the platform instance in Figure 1 integrates 2 *synapse* blocks per unit, which means that each synapse block calculates the contribution given by 128 neurons. The outputs of the of *synapses* blocks are summed-up by an *adder-tree* module to produce *I*. The interactions with the *weight RAM* block and the synchronization with the *Neuron Section* block is controlled by a dedicated FSM, the *synapses-FSM*.

#### 2.2.2. Units: execution flow

The execution flow of a *unit* within each simulation cycle can be described as follows:

1. According to the currently processed neuron index, the *neuron_FSM* drives the *AddGen* block, which generates the address *Ad*(*k*), used to correctly fetch the current *v*(*k*) and *u*(*k*) values from the *u-v RAM* block, and to retrieve the IZ parameters from the *parameter RAM* blocks;
2. The *Izhikevich* block executes all the multiplications in Equations (4) and (5), the *Synapses Section* determines *I*. The *neuron_FSM* and the *synapses_FSM* exchange control signals to synchronize these two operations, in order to make sure that the proper value *I* is added in Equation (4);
3. As already said, *I* is a weighted sum, depending on the firing neurons. The *synapses_FSM* drives the *weight AddGen* to correctly access the *weight RAM* that stores the *synaptic weights* of the excitatory and inhibitory neurons. *Ad(weights)* represents the addresses of the memory locations storing the weights associated to the excitatory and inhibitory neurons connected to the currently processed one;
4. As soon as Equations (4) and (5) are completely computed, the *neuron_FSM* enables the possibility of writing back the updated *v*(*k* + 1) and *u*(*k* + 1) values on the *u-v RAM* block. The *neuron_FSM* drives the *AddGen* to access the correct location of address *Ad*(*k* + 1);
5. The final processing stage of each single neuron requires to evaluate whether the spike has to be fired or not. The result is written in the correct position (determined by the currently processed neuron index) of the *out_spike_reg* module.

All these steps are iterated for all the neurons within the *unit* to evaluate and update the status of the SNN. The *neuron_FSM* is responsible of verifying whether all the neurons have been processed or not and to notify that the content of the *out_spike_reg* module is the complete updated *unit* contribution to the neuronal activity that can be sent to the *concatenation logic*. Depending on the initialization data stored within *u-v RAM, parameter RAM* and *weight RAM* blocks, the platform is thus capable to fully emulate an arbitrary IZ-based SNN.

A fixed-point arithmetic was adopted in order to pursue a fast execution and a compact hardware implementation. A Q3.4 format for the weights (meaning that a Q format is adopted, with the decimal point virtually placed in order to leave 4 bits for the fractional portion over a 7-bit word) was chosen. Such a solution limits the dynamic ranges of the weights with minor effects, since the low values are usually swamped by the thalamic noise term (Thomas and Luk, 2009). A more conservative approach based on the worst-case design, which is unrealistic in this kind of applications, would limit the SNN weights in the [−1,1) range. Multiple iterations with NEST have been performed in order to define a format for the weights able to reduce the memory requirements while preserving an acceptable accuracy. In order to avoid intermediate overflows, the synapse accumulators use 8 bits for the input and 32 bits for the outputs, and they are connected to a 32-bit in 32-bit out adder tree. Both of them are implemented using DSP48 macros for Xilinx FPGAs. In order to be able to represent the IZ parameters with enough flexibility to accommodate typical models and novel ones, a Q10.22 was selected for them. The internal computations rely on pre-computed constants, full-precision adders and multi-cycle multipliers with 32-bit in and 32-bit out.

The communication between the emulation platform and the external environment is required, at start-up, to allow the pre-loading of neuron parameters and synaptic weights for the target experiment inside the system memory blocks. Moreover, emulation results must be sent to the external environment, respecting real-time constraints, during the whole emulation time. Communication interface can be implemented in different ways, exploiting the connectivity in modern FPGA boards and the support offered by design tools and programming environment. Two different communication methods were tested. Firstly, the exploitation of a host general-purpose processor, implemented as a Microblaze soft-core on the FPGA, was evaluated. The processor reads system memories as part of its memory map and communicates with the external environment using an Ethernet connection or a serial UART connection. This solution is easy to implement and provides comfortable coupling of the processor with the emulator, but presents the disadvantage to occupy resources on the reconfigurable device, which may be exploited to emulate more neurons. A second solution is available using FPGA families that embed hard-wired processing cores, such as the Xilinx Zynq family, which includes chips integrating programmable logic and an ARM dual-core processor. In this case, an interface between the emulator, implemented on the programmable logic, and the ARM sub-system, serving as a host processor and providing adequate connectivity with the external environment, was developed. A master IP that loads ad stores data on the DDR memory connected to the ARM when needed, exploiting a set of 4 AXI-based interfaces natively available in the system, was mapped on the FPGA. Each of the AXI interfaces provides a bandwidth of 64 bits/cycle and can be clocked at more than 100 MHz, thus is sufficient to sustain output of emulation results in real time.

However, it must be noticed that the best interface implementation depends on the target FPGA device and on the target use-case of the emulation infrastructure.

### 2.3. Platform validation approach

#### 2.3.1. Architectural performance evaluation metrics

The main performance evaluation metrics for the FPGA-based hardware implementation are timing and resource utilization. In the proposed parametric architecture, both depend on the chosen configuration of the architectural parameters, i.e., the number of units, the number of synapse modules inside each unit and the precision of the weights. The main limiting factors for the proposed implementation are the DSP modules and the RAM modules. In particular:

- DSP48E1 modules are computing modules used to implement *add* and *multiply* operations,
- RAMB36E1 modules are RAM memory macros that are used to implement memories in the architecture. Each macro has a capacity of 32 kbits.

Obviously, the amount of DSP48E1 and RAMB36E1 needed to implement a given configuration is dependent on the architectural parameters. Performance will be evaluated considering the maximum number of neurons that can be emulated in real-time by the platform. This metric is impacted by several factors:

- real-time constraint,
- architectural parameters,
- implementation level variables (such as working clock frequency and FPGA resource utilization).

In order to provide an overview of the involved dependencies, a model, described in Section 3.1.1, was developed to enable a prospective user to estimate the achievable performance level for different architectural configurations implemented on different FPGA devices.

#### 2.3.2. Accuracy evaluation tests

To evaluate the accuracy of the simulations performed using the hardware SNN, a sister-pool of simulations with NEST (Gewaltig and Diesmann, 2007) was performed. In this way, it was possible to compare the so generated dynamics of the *in silico* (implemented in NEST) and hardware SNN, by means of well-known statistics commonly used to analyze the electrophysiological activity of large-scale neuronal networks coupled to Micro-Electrode Arrays (MEAs). For this purpose, an heterogeneous 1,024-neuron SNN with 768 excitatory neurons and 256 inhibitory ones was simulated. Compared to the maximum number of synthesizable neurons, which is 1,440, this number represents the highest power of two and was chosen in order to simplify the scripting operations required, for the time being, to load the parameters and analyze the results. The DC input currents are 4 pA and 2 pA for excitatory and inhibitory neurons respectively; neurons are randomly connected, according to the generation model in Izhikevich (2003); inhibitory neurons have stronger synaptic connections. In order to achieve heterogeneity (i.e., to model all exctitatory and inhibitory neurons), excitatory cells are generated by assigning (*a*_*i*_, *b*_*i*_) = (0.02, 0.2) and $\left(c_{i},d_{i}\right)=\left(-65,8\right)+\left(15,-6\right)r_{i}^{2}$, where *r*_*i*_ is a random variable uniformly distributed on the interval [0,1] and *i* is the neuron index; similarly, each inhibitory cell has $\left(a_{1},b_{i}\right)=\left(0.02,0.25\right)+\left(0.08,-0.05\right)r_{i}^{2}$ and (*c*_*i*_, *d*_*i*_) = (−65, 2).

To characterize the spiking activity, the mean firing rate (MFR) of the network and the inter spike interval distribution (ISI) of the excitatory and inhibitory neurons were evaluated. The bursting activity was characterized by means of the mean bursting rate (MBR), burst duration (BD), and inter-burst interval (IBI). Bursts have been detected by using the algorithm devised in Chiappalone et al. (2005). Detected bursts are sequences of spikes having an ISI smaller than a reference value (set at 100 ms in our simulations), and containing at least a minimum number of consecutive spikes (set at 4 spikes in our simulations). Briefly, MFR and MBR are the number of detected spikes per second and bursts per minute falling in a temporal window equal to the duration of the simulation. The ISI and IBI distributions are the probability density functions of time intervals between consecutive spikes and bursts, respectively (Dayan and Abbott, 2001). Finally, BD is the duration of the detected bursts.

In addition to the aforementioned statistics, the spike jitter between *in silico* and hardware simulations was also computed. Practically speaking, by considering as reference the spike timing of the NEST simulations, the temporal distance of the correspondent spikes generated by the hardware SNN was computed.

The evolution of the membrane potential of a single neuron in the two simulations were also compared. Five experiments were performed, in order to analyze the dynamics of the *u* and *v* potentials of a modeled neuron respectively belonging to three classes of excitatory neurons (*regular spiking, intrinsically bursting* and *chattering*), and two classes of inhibitory neurons (*fast spiking* and *low-threshold spiking*). The *a, b, c* and *d* parameters belonging to each of the emulated cortical cells are reported in Table 1; the DC input current, for all considered experiments, is 4 pA. The behavior of the emulated potentials with the potential evolution obtained by means of a NEST simulation were also compared.

**Table 1:** **Parameters of emulated cortical cells**.

	***a***	***b***	***c***	***d***
RS	0.02	0.2	−65	8
IB	0.02	0.2	−55	4
CH	0.02	0.2	−50	2
FS	0.1	0.2	−65	2
LTS			−65	2

## 3. Results

In this section, the results have been organized in order to discuss at first those related to the architectural performance evaluation and then those related to the accuracy evaluation.

### 3.1. Architectural performance evaluation results

As previously mentioned, the assessment of the quality of the proposed architecture has to consider two main factors: resource utilization and emulation performance. In the following, a description of the timing and utilization figures that can be obtained changing the architectural parameters is presented. At first, the timing characteristics of the architectures modules and their dependence on the selected architectural parameters have to be studied, within the real-time constraints imposed by the emulation. Then, the optimal parameter values have to be selected, in the range actually allowed by the target FPGA device. All the presented results were verified after synthesis and implementation.

#### 3.1.1. Timing characteristics of the architecture modules

The real-time constraint to be considered defines how often the SNN output has to be evaluated. Such a metric, in emulation/simulation, is usually referred to as *delta cycle*. A delta cycle of 0.1 ms was chosen, aiming to be aligned with an acceptable sampling frequency in the scope of acquisition of signals from neuronal cultures with MEAs. The same time step was also used for the differential equations discretization, hereafter called *T*_*sample*_.

As mentioned, hardware structures are reused to emulate more neurons, in the considered interval. Emulation of one neuron occupies the set of hardware resources in a unit for a determined number of cycles, that will be indicated in the following as *T*_*emu*_. In order to relate *T*_*emu*_ with the real-time constraint, actual clock period, which will be indicated as *T*_*clock*_ in the following, used as synchronization reference within the architecture, shall be considered. In synchronous digital systems, the minimum clock period that can be chosen by the designer is related to the propagation delay of gates implementing combinational paths through the design. In the design of the proposed architecture, a pipeline strategy that allows the minimum clock period to be independent from the architectural configuration was adopted. After the implementation on the FPGA device, the optimal value of *T*_*clock*_ can be evaluated to be 10 ns, corresponding to a maximum working frequency of 100 MHz. It was proven that this frequency can be substained for all the configurations that may fit in mid-to-high end FPGA devices, confirming the scalability of the proposed architecture.

Thus, the number of cycles available for emulation in a sampling interval is *T*_*sample*_/*T*_*clock*_ = 10, 000 cycles. Then, each unit can emulate *N*_*neu*_ neurons in one sampling interval, where

(6) $$\begin{array}{l} N_{neu}=10,000/T_{emu} \end{array}$$

*T*_*emu*_ is a function of the number of synapse modules *N*_*syn*_ and units *N*_*units*_ in the system. The number of cycles needed to emulate one neuron is the sum of two contributions:

(7) $$\begin{array}{l} T_{emu}=log_{2}\left(N_{syn}\right)+\frac{N_{neu}*N_{units}}{2*N_{syn}} \end{array}$$

Equation (7) was constructed on the basis of the architectural details of the hardware modules and its validity was verified in HDL-level simulation and after actual implementation. The first contribution is an offset related to the pipeline stages in the adder tree connecting the output of the synapse modules. The second contribution is the actual time needed to perform all the accumulation routine that calculates the synaptic current. In the second term the numerator represents the total number of neurons constituting the emulated network, the denominator takes into account that the workload related with the accumulation is divided between the synapse modules in the unit, each one performing two *add* operations per cycle.

Combining Equations (6) and (7), *N*_*neu*_ can be calculated solving a quadratic equation, on the basis of the architectural parameters *N*_*syn*_ and units *N*_*units*_. Eventually, the total number of neurons that can be emulated is *N*_*units*_ × *N*_*neu*_.

**Figure 4:**
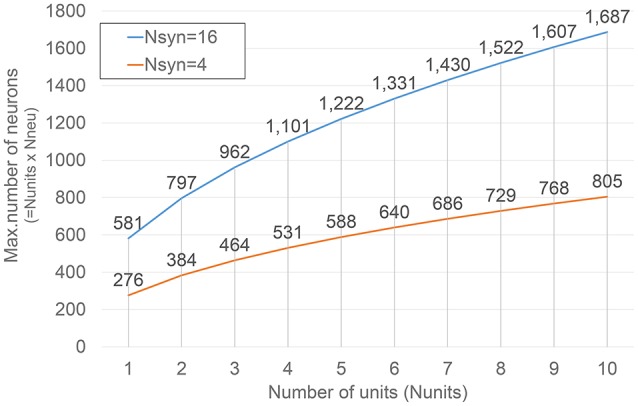
**Dependence of the maximum number of neurons under emulation on ***N***_***units***_**. Results corresponding to two different numbers of synapse modules (*N*_*syn*_) are presented.

Figure 4 shows how such numbers changes varying *N*_*units*_ for two different *N*_*syn*_ values. The selection of the values for such parameters is obviously bounded by the amount of resources available on the target FPGA device, as it will be described in more detail in the next section.

Even though the proposed architecture was not conceived as an hardware accelerator but, rather, a real-time simulation platform enabling closed-loop neurophysiological experiments, a rough performance comparison against NEST can be presented. When running on a PC platform (Ubuntu 14.04 LTS, kernel 3.13.0-96-generic, CPU Intel(R) Core(TM) i7-2600 CPU @ 3.40 GHz, 16 GB RAM), the emulation of the SNN with 1,440 neurons requires 6.3 s per second of simulation, which is far from the real-time achieved with the proposed architecture. Nevertheless, caution should be used when considering such numbers for a fair comparison. In fact, on one hand, the architecture is clocked in order to provide the real-time performance at 10 kHz, so the actual processing time is masked by the (higher) wall time, which is 0.1 ms. The same does not hold for a PC-based emulation, not constrained by such a wall time. On the other end, different programming styles, programming language and processor architectures can lead to very different results, so the performance of PC-based solutions could seem unfairly poor.

#### 3.1.2. Hardware resources utilization

In order to select the correct architectural parameters, it is mandatory to understand their impact on the utilization of hardware resources in the target device.

When the architectural configuration is known, it is very easy to estimate the number of required DSP48E1 macros. Each unit uses 3 macros for each synapse module and 3 macros to implement the hardware emulating the neuronal dynamic. The amount of RAM macros depends on the total number of neurons to be emulated. The architecture should embed enough storage to contain all the weights determining the contribution of each pre-synaptic neuron to the post-synaptic ones. Such neurons are prospectively disjointed, thus the utilization of resources roughly has a quadratic dependence on the size of the emulated network. Some memory resources are also needed to store neurons' parameters and evolving values of *u* and *v*.

Considering the number of resources in commercial FPGA devices, for typical network configurations, the limiting factor is very often the availability of Block RAM (BRAM). This can sometimes limit the maximum number of neurons under emulation with respect to the possibilities offered by a given parameterization of the unit hardware modules.

In order to provide an estimation of the architecture configurations that may be implemented on mid-range commercial devices, the hardware-related features of a configuration featuring 8 units, each one embedding 16 synapse modules, realized on a XC6VLX240T Xilinx device, are presented. Table 2 represents the device utilization summary.

**Table 2:** **Summary of the resource utilization**.

	**Used**	**Available**	**Utilization**
Registers	48,502	301,440	16%
LUTs	55,884	150,720	37%
RAMB36E1	392	416	94%
DSP48E1	408	768	53%

*Target FPGA platform is a XC6VLX240T Xilinx device. The implemented architecture features 8 units with 16 synapses, capable of emulating 1,440 neurons*.

This configuration occupies 94% of the BRAM resources and 53% of the DSP48 macros, and it is capable of emulating 1,440 neurons in real time.

Although power consumption analysis has been considered as a secondary development objective in this work, it is worth to provide some hints about the power-related features of the proposed architecture. Obviously, the actual power dissipation depends on the specific FPGA platform selected as target. However, for every FPGA device considered in the developed experiments, a significant part of the power consumption is related to the usually called quiescent power, that is the power dissipation of the idle FPGA, before its actual programming. Moreover, recent all-programmable FPGA-based SoCs as Xilinx Zynq devices, present an additional contribution to the power consumption due to the host processing system implemented on the chip. Finally, in general FPGA chips are mounted on a development board including several peripherals, that add a further contribution unrelated with the emulation. Thus, the power consumption of the overall emulation platform is weakly dependent on the number of emulated neurons. As an example, in the presented experiments, a Xilinx ZC706 evaluation board featuring a XC7Z045 FPGA chip dissipates 6.8 W in the idle state and 8.5 W when executing emulation with a 100 MHz clock on the FPGA.

### 3.2. Accuracy evaluation results

#### 3.2.1. Single neuron membrane potential evolution

**Figure 5:**
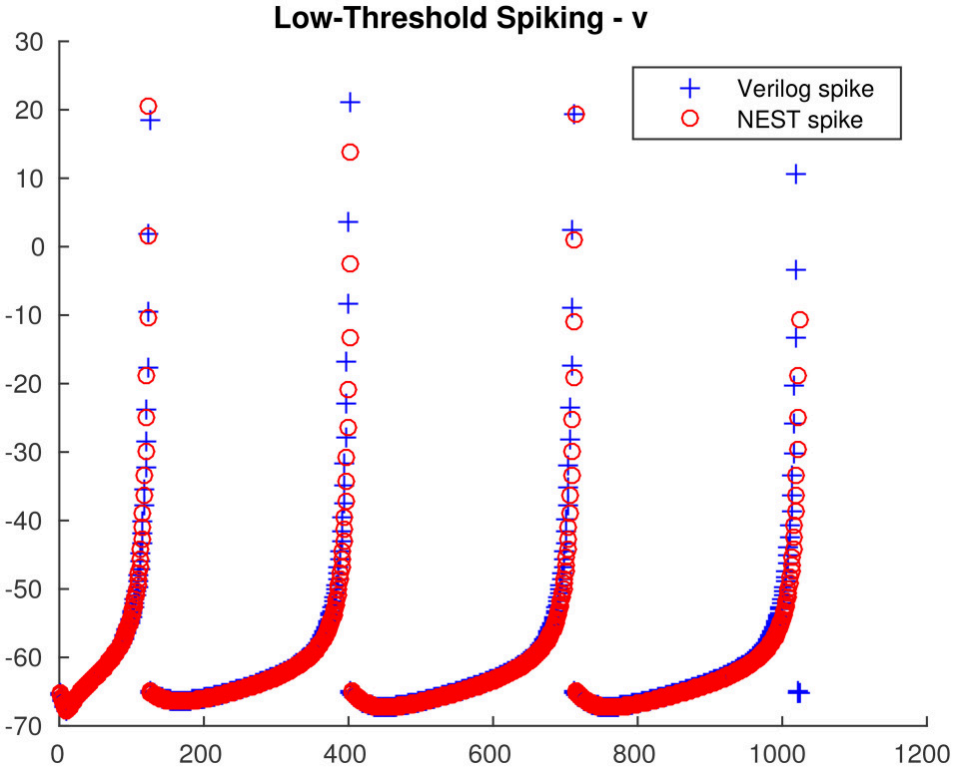
**Low-threshold spiking neuron membrane potential evolution: comparison between the proposed architecture and NEST**.

**Figure 6:**
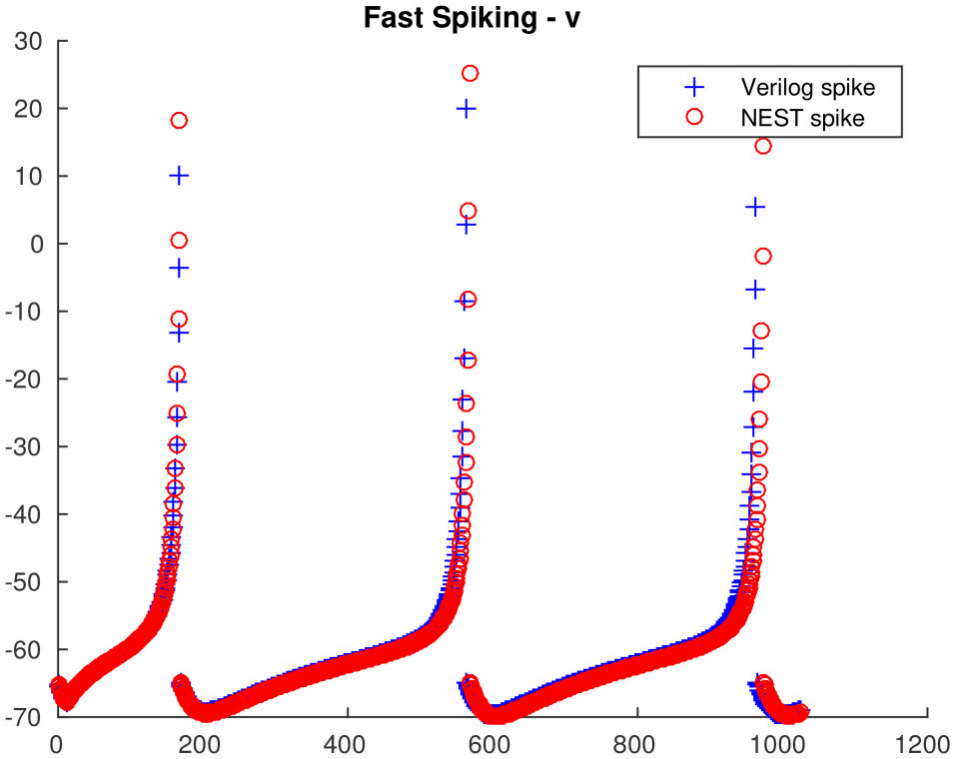
**Fast spiking neuron membrane potential evolution: comparison between the proposed architecture and NEST**.

From the single neuron simulation, it is possible to find a good adherence between the membrane potential evolution as obtained with the proposed architecture and with NEST. This is clearly visible in Figures 5, 6 respectively for low-threshold spiking and fast spiking neurons.

#### 3.2.2. Fully-connected network of N neurons

**Figure 7:**
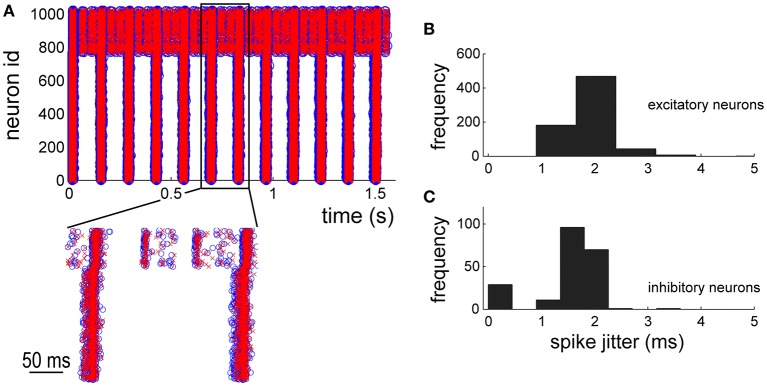
**Network dynamics characterization: comparison between ***in silico*** and hardware simulations. (A)** 2 s of electrophysiological activity. Blue circles and red crosses are representative for software and hardware approaches, respectively. **(B,C)** histograms of the spike jitter between software and hardware simulations evaluated for excitatory **(B)** and inhibitory **(C)** neurons of the network.

By using the NEST simulations as reference, the electrophysiological patterns of activity generated by the hardware implementation of the network model were compared. The raster plot of Figure 7A shows the spike timing of the 1024 neurons of the network. The first neurons (id: from 1 to 768) are excitatory whereas the others inhibitory (id: from 769 to 1024). Blue circles and red crosses are representative for the two simulation approaches (i.e., software, blue circles, and hardware red crosses, respectively). The zoom of Figure 7A shows a good overlap of the spike timing. In order to quantify such a jitter, the histogram (bin width equal to 0.3 ms) of the occurrences relative to the excitatory (Figure 7B) and inhibitory neurons (Figure 7C) was plotted. Both the neuronal populations display significant jitters less than 2.0 ms in 95% of the occurrences, indicating good performances of the hardware implementation of the network model.

**Figure 8:**
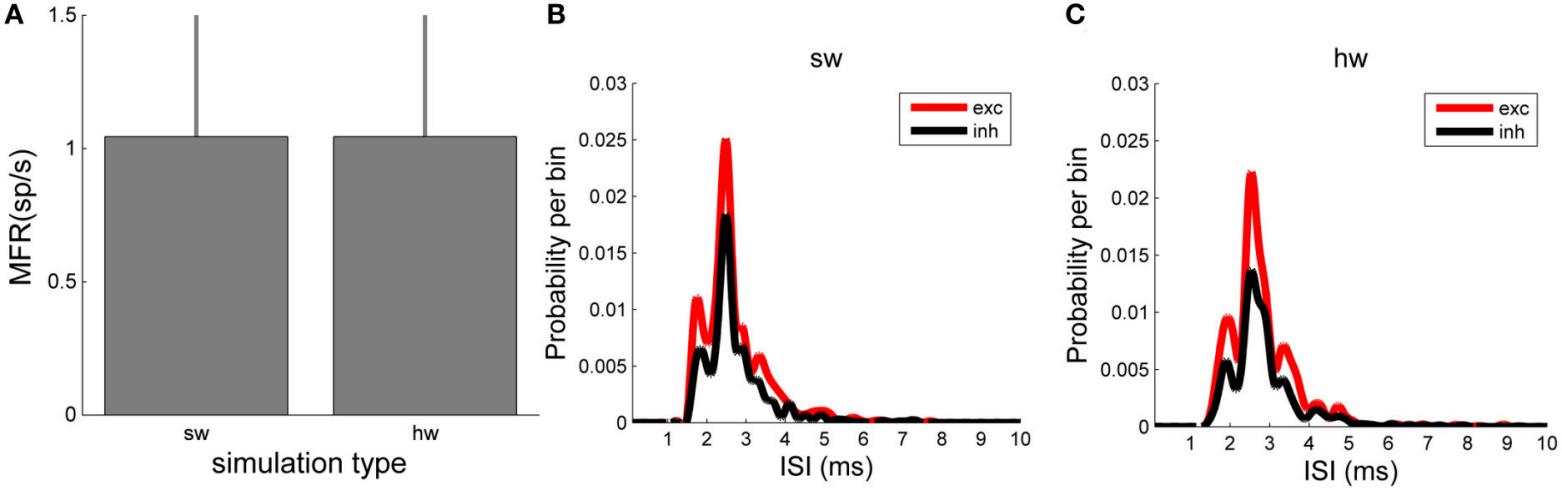
**Network dynamics characterization: comparison between ***in silico*** and hardware simulations. (A)** MFR (evaluated over the whole neurons of the network). No statistical difference can be evaluated between the two simulation approaches. **(B,C)** ISI distributions of the excitatory (red) and inhibitory (black) neuronal populations evaluated in the *in silico* **(B)** and in hardware **(C)** simulations: the same trend can be appreciated.

In terms of number of spikes, both the software and hardware models present the same number of spikes, as the plot of the MFR displays (Figure 8). MFR values are 1.05 ± 0.48 spikes/s in the NEST implementation of the model and 1.05 ± 0.49 spikes/s in the hardware one. Finally, the ISI distributions (Figure 8B, relative to the software, and Figure 8C, relative to the hardware model) by splitting the contribution of the excitatory (red line) and inhibitory (black line) populations, were evaluated. This analysis shows a good agreement of the two model implementations too. Qualitatively, the shape of the ISI distributions is similar, as well as the temporal position of the peaks of the curves (hardware: 2.54 ms vs. software: 2.50 ms).

**Figure 9:**
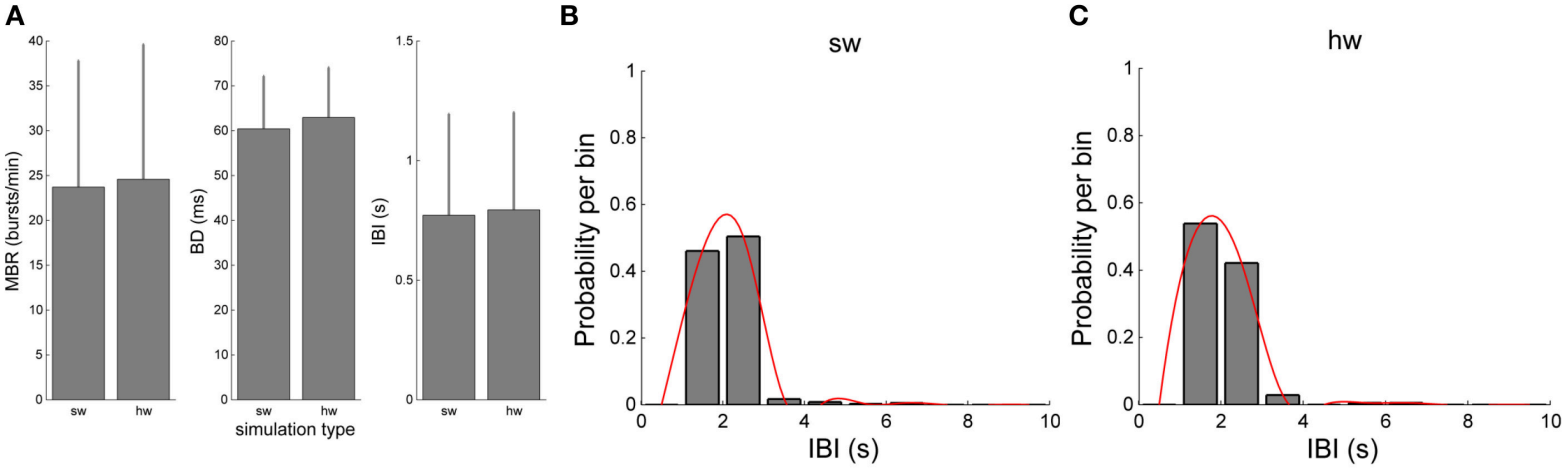
**Comparison of the bursting features of the simulated network by using the software and hardware approaches: (A)** MBR, BD, IBI **(B,C)** IBI distributions.

The validation of the hardware network model was carried out by comparing the bursting dynamics (Figure 9). Figures 9A–C compare the values of MBR, BD, and IBI of the software and hardware network models, respectively. Although slight differences can be appreciated, such differences are not statistically significant (*p* >0.05, Mann-Whitney, non-parametric test). The Mann-Whitney *U*-test was chosen since the analysed data do not follow a normal distribution, as revealed by means of the Kolmogorov-Smirnov normality test applied to them. The chosen *p*-value is assumed to be adequate for the considered problem. Similar considerations can be done for the IBI distributions (Figures 9B,C).

## 4. Discussion

Several SNN hardware accelerators have been proposed so far in the scientific literature (Maguire et al., 2007). They usually try to overcome the performance limitations of purely software simulators (Brette et al., 2007) such as NEURON (Carnevale and Hines, 2006), NEST (Gewaltig and Diesmann, 2007), BRIAN (Goodman and Brette, 2009), etc., widely accepted as research tools in the community of computational neuroscience. However, unless an explicitly parallel processing is pursued on large multiprocessors high performance computing platforms, such simulators suffer the intrinsic scalability limits of the underlying object of the simulation, becoming soon too slow for large-scale networks of biologically plausible neuronal models (Rast et al., 2010). In this section, a discussion about some relevant architectures for SNN simulations in hardware is presented, along with a comparison with the proposed approach. Among the different architectures cited hereafter, those aimed to simulate a large number of neurons have been grouped in Table 3, reporting the main relevant data gathered from the cited sources. Without being exhaustive, this table enables a quick overview of the present state of the art.

**Table 3:** **Summary of some relevant state-of-the-art architectures for SNN hardware simulation**.

**Work**	**Target**	**♯Neurons/core**	**♯Cores**	**Model**	**♯Synapses/neuron**	**Time res**
Schoenauer et al., 1998	ASIC	≈ 30,000	4	LIF	> 30	< 1 *ms*
Wolff et al., 1999	Multi-processor (DSP)	≈ 1,900	64	Eckhorn	> 30	> 1 *ms*
Glackin et al., 2005	FPGA	≈ 1,000	4	I&F	≈ 500 *k*	−
Upegui et al., 2005	FPGA	30	1	custom	30	≈ 1 *ms*
Pearson et al., 2007	FPGA	112	10	LIF	≈ 912/112	0.5 *ms*
Cassidy et al., 2007	FPGA	51	1	LIF	128	320 ns
Jin et al., 2008	Multi-processor (ARM)	1,000	1	IZ	100	1 *ms*
Thomas and Luk, 2009	FPGA	1,024	1	IZ	1,024	10 *μs*
Ambroise et al., 2013	FPGA	117	1	IZ	117	1 *ms*
Cheung et al., 2016	FPGA	> 98,000	6	IZ	1, 000 − 10, 000	1 *ms*

*For some architectures flexible enough to implement several neuronal models, only one published result is reported*.

Despite the purely software solutions present the aforementioned limitations, it is obvious that they usually pursue simplicity (in the creation and simulation of the model), precision (typically double) and flexibility (the possibility to change topology, parameters, synaptic model, neuron model, etc.). For this reason, when moving toward the hardware simulation systems, it is obvious the success of architectures exploiting efficient signal processing cores, such as ParSPIKE (Wolff et al., 1999), which is based on the Analog Devices ADSP21060 Digital Signal Processor. In fact, Digital Signal Processors revealed better performance than high-end mainstream processors in several biomedical and signal processing applications, with a power consumption that could be even two orders of magnitude lower (Pani et al., 2013, 2014) and they are currently being used for studies in neuroprosthetics (Pani et al., 2011, 2016).

A very successful implementation of a hardware architecture for SNN based on general purpose (embedded) processors is SpiNNaker (Furber et al., 2014). It is a multilevel tiled architecture, i.e., an architecture composed, at different levels, of a regular mesh of computing elements called tiles, mixing the flexibility of a software implementation of the neuronal model with the performance of a custom architecture (at macroscale). The smallest tile is a node, i.e., a custom chip consisting of multiple (up to 18 in the latest versions) ARM968 processors clocked at 200 MHz, without embedded floating-point units and exploiting a network-on-chip infrastructure for the communications. These nodes are assembled in boards comprising 48 of them, and exploiting highly customized self-timed connections. These boards communicate each other through custom serial links implemented on FPGA. This architecture is being used in the human brain project (www.humanbrainproject.eu). Despite the impressive work behind this platform, it is neither suited for a neuroengineering lab with limited budget nor for closed-loop applications. Furthermore, compared to our design, each core is able to model up to a few hundred neurons (LIF or IZ) with about one thousand input synapses each. The time resolution scales down to 1 ms only, which is inadequate for interfacing with the living tissue. Recent completely asynchronous extensions of the software framework on the SpiNNaker platform allowed performing simulations with networks implementing sub-millisecond tasks, such as sound localization, (Lagorce et al., 2015). However, some specific conditions apply, as the use of even-driven LIF neurons, the adoption of a dendritic delay core for every particular delay value (leveraging the large number of cores available), etc.

The same holds for the NeuroFlow architecture, which for sure represents the state of the art in the field of FPGA architectures for SNN (Cheung et al., 2016). Compared to it, the proposed architecture targets a real-time performance of 0.1 ms, which can be ever reduced by changing the size of the network or speculating on the connectivity (assumed to be full in our tests). In particular, the time step of 0.1 ms is one order of magnitude less then that of the NeuroFlow architecture. Furthermore, the impressive numbers of simulated neurons provided by NeuroFlow (up to 600,000 units) can be reached with 6 FPGAs, with a toroidal network configuration, limiting the number of synapses to 1,000–10,000, when the connection probability follows a Gaussian probability of the synaptic distance with standard deviation ranging from 32 to 512. This unfortunately makes a point comparison hard and potentially unfair. Overall, NeuroFlow targets larger networks than the proposed architecture, with a different aim which is closer to that of SpiNNaker. This is reflected by the choice of a large off-chip Dynamic Random Access Memory (DRAM) compared to the BRAM used in our and other designs, and by the higher flexibility in the simulation setup.

On the other end of the flexibility axis, it is possible to find neural architectures based on application-specific integrated circuits (ASIC). They usually range from the analog neuromorphic chips (Hofstoetter et al., 2005), which exploit the possibility to make the transistors work on the current flow as the ion channels do on the ions flow, to the custom VLSI digital neurocomputers (Van Sickle and Abdel-Aty-Zohdy, 2009). Typically, the former are more complex to design but achieve better performance than the latter, both in terms of silicon area and power consumption (Joubert et al., 2012). In order to take the best in terms of performance while preserving the flexibility of software solutions, programmable hardware progressively gained interest in the computational neuroscience community. If field programmable analog arrays are still not completely convincing in terms of performance, even though some reconfigurable analog VLSI neuromorphic chips exist (Yu et al., 2010), on the digital side there is a growing interest toward the use of FPGA for these purposes (Maguire et al., 2007). FPGAs, providing the user the possibility to reconfigure the device by full or partial reload of the configuration bitstream, present the advantages of a custom architectures (as for the ASIC) and a flexibility approaching that of software implementations. Furthermore, the presence of IP cores enables the creation of multiprocessors systems on chip even on FPGA (Glackin et al., 2005).

In the largest part of cases, the effort toward the development of very fast architectures had a negative impact on the biological plausibility of the adopted neuronal model. In fact, the neuronal models that have been presented in the scientific literature so far are characterized by different biological plausibility and computational complexity (Paugam-Moisy and Bohte, 2012). Despite even the most complex Hodgkin-Huxley model was implemented in hardware (Graas et al., 2004), not all of them are suited for medium to large scale SNNs on digital hardware, because they should be computationally simple and at the same time capable of representing the wide variety of firing patterns exhibited by the different biological neurons. For this reason, some of the architectures use simplified custom neuronal models (Upegui et al., 2003, 2005), much more use integrate-and-fire (I&F) (Glackin et al., 2005) or leaky-integrate-and-fire (LIF) (Cassidy et al., 2007; Pearson et al., 2007). A recent interesting investigation on the limits of computer based approaches, FPGAs and Graphics Processing Units (GPUs) on highly complex biologically-plausible models of neurons belonging to the cerebellar cortex was reported in Florimbi et al. (2016). This work, in the main framework of the Human Brain Project, remarks how such complex neuronal models require huge hardware resources so that single-chip FPGAs cannot be an effective platform for cell networks, even though ASICs could, whereas GPUs can provide speedups that are still far from the real-time bound.

The proposed architecture exploits the very efficient IZ model (Izhikevich, 2003). Other works at the state of the art implemented the same neuronal model. Usually, for computational complexity reasons, the fixed-point processing is preferred. Despite this approach obviously limits the precision of the operations, compared to a floating-point solution, it has been shown to be adequate for several practical applications. First thing to notice is that several papers in the past described the implementation of the IZ model on FPGA without embedding it into a SNN. For instance, in Cassidy and Andreou (2008) a hardware implementation on the neuronal model alone is described (the model equations were changed in order to exploit power-of-two arithmetic, leading to less precision). The absence of the synapses is a remarkable limitation because of the quadratic dependence from the number of neurons of the synaptic weights, which is the main issue for scalability.

Other works, such as Rice et al. (2009), even though introducing the synapses, present topologies such that synapses connectivity is a minor issue. In that case, for instance, the number of neurons in very high (about 96 × 96, as much as the pixels of the input images) but the synapses are not as much as the square of such a value but rather only 48 times it. In that case, a Q4.12 format was used for the parameters, with the weights represented in Q4.12 format. The format depends on a trial and error procedure. SpiNNaker, for instance, uses different scaling factors for different parameters and values, showing that the best results can be achieved with a Q8.8 format for *u*, *v*, *c*, and *d*, whereas the Q0.16 format was chosen for parameter *a* and *ab* (since *b* alone is not used in that implementation), limiting such parameters to be <1 (Jin et al., 2008). With such an approach, the architecture is able to simulate up to 1000 IZ neurons on a single fascicle in a network with a low connectivity level (10%). Nevertheless, such a low connectivity is unrealistic: in Thomas and Luk (2009) a connectivity with 1,000 synapses per neuron, claimed to be a common-sense choice, is simulated. In this case, the fixed point representation used for the weight and the arithmetic of the adder tree is used, due to the limits imposed by the memory limits of the chosen platform, and weights are limited to 9 bits. Our architecture, with its 7 bits for the weights with a Q3.4 format, follows a similar approach, considering that on a physical platform it is acceptable to fix limits to the range of such parameters (Thomas and Luk, 2009). Spikes accumulation is performed at 32 bits to preserve as much as possible the precision. In Ambroise et al. (2013), the authors present a similar approach, that is capable of emulating up to 167 neurons (it is worth to notice that these results have been achieved on a smaller device: no data is provided on large FPGAs). Compared to the proposed one, such a work uses a higher number of resources due to higher data precision and to a fairly more complex processing of the synaptic current. It also considers a reduced sampling frequency (1 kHz) with respect to our work. In Glackin et al. (2005), the I&F model is implemented in fixed point with a Q8.10 precision for the membrane voltage and 12-bit precision for the synaptic conductance (no further details on the data size), using powers of 2 for the scaling parameters in the model, to avoid multipliers and dividers, with some precision loss.

In the proposed work, precision of the computation is demonstrated by the achieved results. The architecture is capable to obtain the same firing patterns of NEST, with a real-time performance that reaches 0.1 ms. The slight differences cannot be considered a limiting factor for the exploitation of the architecture in real-time closed-loop experiments, since the global firing patterns are respected. Even though the proposed architecture is not highly optimized, because of the need to ensure flexibility at this development stage, it is possible to pursue energy/performance efficiency by means of fine application-driven customization of the hardware architecture, that requires adequate support by advanced design tools (Jozwiak et al., 2012, 2013). The proposed approach can evolve in such a direction for closed-loop implantable implementations.

In perspective, the possibility to have a valid and efficient hardware tool to simulate and generate in real-time realistic spiking dynamics could pave the way to the design of new devices to interface synthetic neuronal assemblies to biological excitable tissues. Indeed, the so developed architecture could be used to generate realistic signals (in terms of time and spatial constants) to stimulate biological networks (open-loop application) as well as to realize closed-loop systems in which, in a bi-directional way, biological and hardware networks are mutually stimulated. In such scenarios, similar to state-of-the-art closed-loop experiments (Wagenaar et al., 2005; Wallach et al., 2011), the availability of an embedded system implementing in hardware (e.g., FPGA) a biologically plausible SNN would be the only enabling technology. In fact, purely software simulations could not be used to interface *in silico* neuronal models with living beings.

In the meanwhile, the real-time performance of an FPGA platform as the one proposed in this work, overcoming the limitations of the software simulators, can be exploited to study the fundamentals of the interaction between living neuronal assemblies and synthetic ones, in closed-loop, opening to hitherto unexplored neurophysiological experiments.

## Author contributions

All the authors substantially contributed to the manuscript. In particular, PMa, DP, and LR conceived the study, PMa and DP defined the methods and analyzed the performance results, PMe and FP defined the architectural features and coding approach, GT performed the hardware parameterization, debug and co-simulations HDL-NEST. All the authors drafted and revised the manuscript and agree with the current version.

## Funding

The research leading to these results has received funding by the Region of Sardinia in the ELoRA project (Fundamental Research Programme, LR 7/2007, grant agreement CRP-60544).

### Conflict of interest statement

The authors declare that the research was conducted in the absence of any commercial or financial relationships that could be construed as a potential conflict of interest. The reviewer SZ and handling Editor declared their shared affiliation, and the handling Editor states that the process nevertheless met the standards of a fair and objective review.
